# Sustained false-positive results for hepatitis A virus immunoglobulin M: A case report and literature review

**DOI:** 10.1515/med-2021-0336

**Published:** 2021-09-07

**Authors:** Youwen Tan, Li Chen

**Affiliations:** Department of Hepatology, The Third Hospital of Zhenjiang Affiliated Jiangsu University, No. 300, Daijiamen, Runzhou Distinct, Zhenjiang 212003, China; Department of Hepatology, The Third Hospital of Zhenjiang Affiliated Jiangsu University, Zhenjiang 212003, China

**Keywords:** immunoglobulin M, hepatitis A, false-positive

## Abstract

Hepatitis A virus immunoglobulin M (HAV-IgM) is often used to diagnose acute hepatitis A virus (HAV) infection serologically. However, false-positive test results can interfere with the diagnosis. A 56-year-old woman was readmitted to the hospital owing to abnormal liver function tests for the last 18 months. She had been diagnosed with acute HAV and was hospitalized in isolation based on a positive HAV-IgM test 18 months ago. Regular follow-up after discharge showed abnormal liver function and an elevated level of antinuclear antibodies and immunoglobulin G. For the last 15 days, the patient had fatigue, decreased appetite, and yellow urine, signaling recrudescence. Liver function tests were also abnormal. Liver biopsy revealed histological changes consistent with typical autoimmune hepatitis. After 2 months of methylprednisolone treatment, liver function returned to normal, and HAV-IgM turned negative. The diagnosis of acute HAV in nonendemic areas requires a comprehensive analysis of epidemic history, clinical characteristics, etiology, etc.

## 1 Introduction

Immunoglobulin M (IgM) is a marker for acute viral infection and has definitive clinical value. Therefore, physicians depend on the serum marker for the diagnosis of active infection. However, an increased number of false-positive test results can lead to misdiagnosis and incorrect treatment. Relying solely on this test result further compounds the problem. Many healthcare professionals are still unaware of this issue. False-positive IgM results can occur with any pathogen. We report a case of HAV-IgM with persistently abnormal liver function tests that lasted for 18 months. The patient was incorrectly diagnosed with acute hepatitis A and, finally, with autoimmune hepatitis (AIH).

## 2 Case report

**Figure 1:**
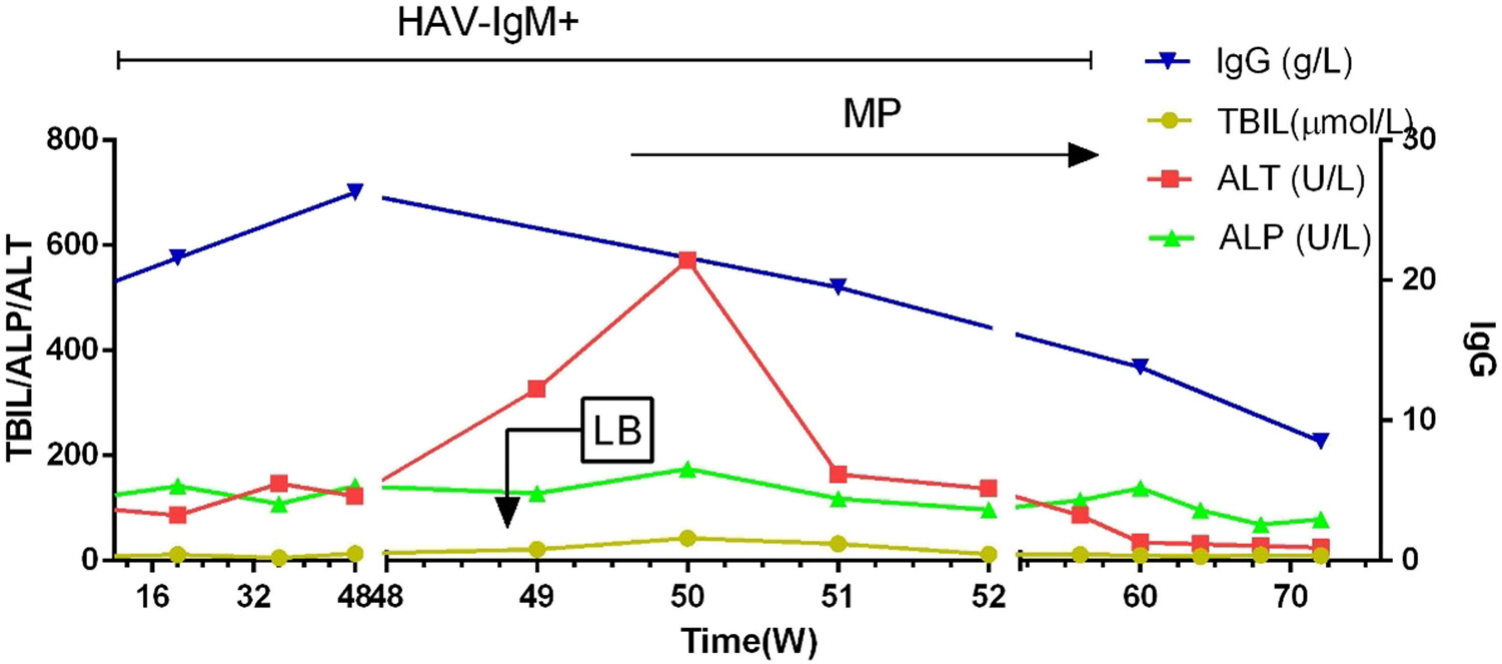
Changes in the liver function and immunological indexes (LB: liver biopsy; MP: methyl prednisolone).

**Figure 2:**
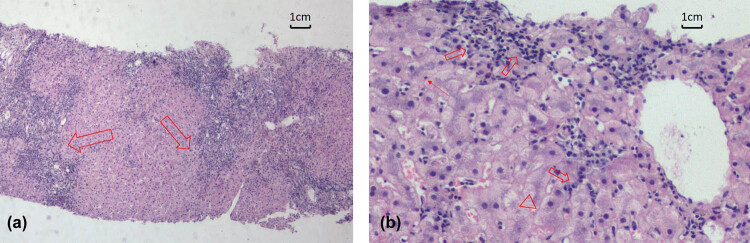
Pathological features of the liver (hematoxylin-eosin staining). (a) Severe interfacial inflammation (thick arrow), ×100 and (b) lympho-plasma cell infiltration (thick arrow), lymphocytes penetrate into the hepatocytes (thin arrow), and rose cluster hepatocytes (arrow), ×200.

A 56-year-old woman was readmitted to the hospital with abnormal liver function tests, persisting for the last 18 months. Her hepatic markers were as follows, tested 18 months ago: alanine aminotransferase (ALT) 113 U/L, aspartate aminotransferase (AST) 104 U/L, alkaline phosphatase (ALP) 124 U/L, and glutamate aminotransferase (GGT) 78 U/L. This was attributed to a positive HAV-IgM (ELISA, OD value of attributed absorbance ≥2.1, determined to be positive). She was diagnosed with acute hepatitis A and hospitalized in isolation for treatment. Abnormal symptoms, such as fever, fatigue, abdominal pain, or yellow urine, were not observed. She was treated with glutathione 0.9 g and glycyrrhetinic acid 150 mg/day for 45 days. The liver function recovered, and the markers were as follows: ALT 56 U/L, AST 43 U/L, ALP 109 U/L, and GGT 65 U/L. However, HAV-IgM was still positive. During this time, anti-nuclear antibodies (ANA) 47 U/L (normal <10 U/L) were also detected. Tests for antimitochondrial antibodies (AMAs) and anti-liver kidney microsomal (LKM) antibodies, and anti-smooth muscle antibodies (SMAs) were negative. The immunoglobulin G (IgG) level was 18.3 g/L (normal range 6.2–16.1 g/L) and the IgM level was 2.71 g/L (normal range 0.98–2.04 g/L). Following discharge, regular monthly reexamination showed abnormal liver function with repeated fluctuations (Figure 1). The patient developed fatigue, decreased appetite, and yellow urine for 15 days, thus indicating recrudescence. Liver function tests showed abnormal results [total bilirubin (TBIL) 43 mol/L, ALT 452 U/L, AST 254 U/L, ALP 175 U/L, GGT95 U/L]. Further testing revealed the ANA level of 102 U/L, IgG 26.3 g/L, IgM 2.71 g/L, and IgA 5.32 g/L (normal range: 0.76–3.9 g/L). The analysis of a liver biopsy specimen revealed histological changes consistent with typical autoimmune hepatitis. There was moderate to severe interfacial inflammation, numerous lympho-plasma cell infiltrates, lymphocytic penetration, and rosette-like hepatocytes (Figure 2). HAV-IgM was positive. To rule out the possibility of HAV infection, a reverse transcription-polymerase chain reaction (RT-PCR) was conducted to detect HAV RNA in the serum. The analysis of the current samples, as well as those from a year ago, was found to be negative. Clinical diagnosis of AIH was made with a false-positive HAV-IgM. Methylprednisolone 32 mg/day was administered, which was tapered to 20 mg/day after 1 month. The liver function tests, done daily, returned to normal after 2 months, following which, methylprednisolone was further tapered to 8 mg/day as a maintenance treatment. On reexamination, the patient was negative for HAV-IgM and HAV RNA. The tests for Epstein-Barr virus (EBV), cytomegalovirus (CMV), herpes simplex virus (HSV), varicella-zoster virus, and adenovirus were negative. There was also no drug-induced liver injury or history of alcohol addiction.

**Ethics statement:** Ethics Statement is not applicable for the case report according to the Medical Ethics Committee of the Third Hospital of Zhenjiang Affiliated Jiangsu University, but Informed consent was obtained from the patient for publication of this case report and accompanying images. The study was conducted in accordance with the Declaration of Helsinki.

## 3 Discussion

HAV is a globally prevalent infectious agent causing intestinal disease. It is mainly transmitted by the fecal-oral route, usually developing as an acute infection followed by self-recovery [1]. HAV-IgM is indicative evidence of an active HAV infection. It appears one week after HAV infection and generally lasts for 3–6 months. HAV-IgM has rarely been detected beyond 1 year [2,3]. Compared to HAV RNA detection, HAV-IgM detection is convenient and fast. It is the primary indicator of current infection with HAV, especially in endemic areas. The accuracy of anti-HAV IgM testing is comparable to HAV RNA detection in the diagnosis of acute hepatitis A [4]. In the absence of a hepatitis A outbreak, a disadvantage of the HAV RNA RT-PCR assay is its high cost as it is not a routine investigation in our country, nor in most other countries. Conversely, it has the advantage of being able to detect acute hepatitis A in its early stages, especially during a hepatitis A epidemic [5]. This will prove to be of great significance to control the further spread of the disease. Another limitation of the HAV RNA assay is that it is susceptible to environmental contamination and is not a genuine test for the HAV virus. However, HAV RNA can be present in the blood for a long time (mean: 79 days) and has also been reported to be present for more than 1 year [6].

The HAV vaccine was included in China’s immunization program from 2008. With the universal vaccination of children, the reported incidence of HAV in China has decreased significantly, from 7.34/100,000 in 2004 to 1.66/100,000 in 2015, thus indicating that the prevalence of HAV has been controlled [7]. HAV-RNA is no longer used as a routine diagnostic test having been replaced by the more convenient and cheaper method of IgM antibody testing. In countries where HAV outbreaks are under control, real cases of HAV are rare. For example, in the United States, 10,735 HAV-IgM samples were collected from Kansas City, Missouri, from January 2007 to December 2010. Among them, 49 HAV-IgM positive test results were found in 35 patients. Finally, only 4 patients were detected with acute HAV (11%) [8]. In conclusion, in areas where HAV is controlled, the diagnosis of acute HAV infection should take into account a comprehensive analysis of epidemic history, clinical characteristics, and laboratory examination [9]. Testing HAV-IgM in individuals with asymptomatic infection or with no history of epidemic disease is not recommended [10]. An investigative report shows that the Department of Health and the Centers for Disease Control and Prevention in the United States conducted a survey on individuals with HAV-IgM positivity from 2002 to 2004 [6]. Those who did not meet the clinical criteria for the case were defined as HAV. The report shows that most of the positive test results did not represent a recent, acute HAV infection. In order to improve the predictive value of positive HAV-IgM detection, clinicians should limit laboratory detection of acute HAV infection to those with typical clinical manifestations or those who have been exposed to suspected hepatitis infection in an environment favoring transmission.

The false-positive IgM mechanism is complex and is the result of comprehensive factors, such as polyclonal B cell activation [11,12], vigorous immune response [11,12], influenza vaccination [13], cross-reactive antibodies [14,15,16], autoimmune disease [17], heterologous reactions to similar viruses [18,19,20], subclinical reactivation of latent viruses [21], interfering substances (e.g., rheumatoid factor) [22,23], and naturally occurring biotin IgM antibodies [24]. In addition to the above, cutoff values being set too low [25,26], faulty reagents [27], technical errors (e.g., overreading weakly reactive bands on immunoblots) [28], low pretest probability [8], and inappropriate testing [6,8] also affect the results.

Although a false-positive HAV-IgM should be common in areas where hepatitis A has been controlled, there is a lack of sizeable data based on population surveys. Our search on PubMed, EMBAS, Web of science, and other databases, using the keywords “False-positive HAV-IgM,” or “False-positive hepatitis A IgM,” revealed a total of 3 cases, with a false-positive HAV-IgM and related conditions [17,29,30]. Two cases of false-positive HAV-IgM were associated with AIH [17,29]; Table 1.

**Table 1:** False-positive HAV-IgM cases reported

Case	Country	Age	Sex	True etiology	LFT	Immunologic test	False HAV-IgM	Potential impact	Report
1	Australia	54	F	Idiopathic scleritis;DILI by infliximab	ALT, 829 U/L; AST, 554 U/L	IgG, 18.4 g/L; IgM, 5.71 g/L; ANA), 1:640	Measured on 2 occasions	Infliximab treatment was discontinued, biochemical improvement within 11 weeks	Elaine Tennant [17]
1	USA	78	F	Congestive heart failure	Mildly abnormal liver enzyme levels, which resolved as her CHF was treated	N/A	Low index, twice the cutoff	Public health investigation, exclusion from adult daycare center	Marie Louise Landry [30]
1	Switzerland	38	F	Autoimmune hepatitis and EBV infection	ALT,424 U/L; AST, 244 U/L	ANA ≥ 1:320; SMA ≥ 1:80	Three times were detected, HAVIgM (+, +, −); HAVIgG (+, −, −)	N/A	Marika Valota [29]

False positives for IgM usually occur under three circumstances. First, diseases with similar clinical presentation, such as infectious mononucleosis, CMV infection, EBV infection, human immunodeficiency virus infection [16,19,20], rubella, measles [31,32], hepatitis A, and hepatitis E viral infection [12,33,34]. However, false positives of HAV-IgM caused by CMV, EBV, and HIV are still reported in individual cases, and the mechanism is thought to be an immune cross-reaction. For example, EBV and HAV may induce autologous production of antibodies against triosephosphate isomerase (IgM anti-TPI)) [35]. Abnormal autoantibody-induced immune damage can lead to hemolysis.

Secondly, when there is an overlap with the etiology of other diseases. Finally, IgM positivity with no corresponding clinical symptoms, biochemical indicators, or histological evidence.

False-positive IgM results can lead to a disproportionate response in the prevention and control of infectious disease, thus delaying the treatment of the original disease. This patient was diagnosed with acute HAV, and this attracted the attention of the disease control department. The residence was disinfected, and the patient was forcibly hospitalized in isolation. This inconvenienced both, her work and her life, in addition to mental trauma. Simultaneously, the treatment of AIH was also delayed to a certain extent. The same confusion was seen in other case reports. A 76-year-old woman with congestive heart failure was diagnosed as having HAV by a community doctor in view of a positive HAV-IgM test, which resulted in her being barred from living in the community [30]. False-positive HAV-IgM leads to misdiagnosis of the original condition, leading to the failure of timely and correct diagnosis and treatment of the primary disease. For example, in this case, corticosteroid therapy was used to control the disease until AIH was confirmed by liver histological examination. In China, if liver function is abnormal, especially if liver enzyme levels are elevated, hepatologists often prescribe dicyclol and other drugs, which have been proven to be beneficial for liver inflammation repair, improvement of fibrosis, and alleviation of liver enzymes [36,37]. Although these drugs are yet to be validated in prospective, randomized, controlled, multicenter studies, no significant side effects have been found with these drugs. Some patients use Chinese herbs, including Schisandra fruit and Chuangcao, to protect the liver [38,39]. These herbs have also been shown to be beneficial for treating liver diseases.

Therefore, even though a positive HAV-IgM test forms the basis for the diagnosis of acute HAV, it should not be accepted as the only confirmatory test, especially when results persist, the patient’s condition is severe enough to require hospitalization, or when there are no epidemiological findings. Instead, wherever possible, the diagnosis should be confirmed by other means.

## Appendix

**Table A1:** Changes of main indexes of liver function and immunoglobulin G

Time (w)	TBIL (mol/L)	ALT (U/L)	AST (U/L)	ALP (U/L)	GGT (U/L)	IgG (g/L)
0	8.3	56	45	109	65	18.3
1	12.7	131.2	89	97	49	
4	7.8	104	77	115	64	
20	11.7	86	48	142	90	21.6
36	5.4	147	112	108	57	
48	13.5	123	94	142	59	26.3
49	21.7	327	227	128	86	
50	43	572	554	175	112	
51	32.5	164.4	107	118	89	19.5
52	12.6	137.1	84	97	57	
56	11.7	86.4	56	115	49	
60	10.6	35.2	28	138	63	13.8
64	8.5	31.6	22	96	38	
68	10.7	28.6	18	69	36	
72	9.4	25.2	23	79	47	8.5

W: week; TIL: total bilirubin; ALT: alanine aminotransferase; AST: aspartate aminotransferase; ALP: alkaline phosphatase; GGT: glutamine transpeptidase.
